# Validation of the Cognition Scale of the Hong Kong Comprehensive Assessment Scales for Toddlers

**DOI:** 10.3390/healthcare13192447

**Published:** 2025-09-26

**Authors:** Cynthia Leung, Kelly W. Y. Lau, Becky M. Y. Chan, Carol K. S. To, Chi-Wen Chien, Catherine C. C. Lam, Florence M. Y. Lee, Stephenie K. Y. Liu

**Affiliations:** 1 Mitchell Institute, Victoria University, Melbourne 8001, Australia; 2 Child Assessment Service, Department of Health, Hong Kong SAR Government, Hong Kong SAR, China; kellywylau@gmail.com (K.W.Y.L.); cpbchan@gmail.com (B.M.Y.C.); clammy2008@gmail.com (C.C.C.L.); florence_lee@dh.gov.hk (F.M.Y.L.); stephenie_liu@dh.gov.hk (S.K.Y.L.); 3 Faculty of Education, The University of Hong Kong, Hong Kong SAR, China; tokitsum@hku.hk; 4 Department of Rehabilitation Sciences, The Hong Kong Polytechnic University, Hong Kong SAR, China; will.chien@polyu.edu.hk

**Keywords:** cognition, assessment, preschool, Chinese

## Abstract

**Background/Objectives**: This study aimed to examine the psychometric properties of the Cognition Scale of the Hong Kong Comprehensive Assessment Scales for Toddlers (HKCAS-T) including its measurement properties, concurrent validity, and reliability. **Methods**: Participants included 282 children aged 18 to 41 months. These children were assessed on the HKCAS-T and the Cognitive Scale in the Cognitive Battery of the Merrill-Palmer-Revised Scales of Development (M-P-R). For test–retest reliability, 41 children were reassessed four weeks after the initial assessment. **Results**: Rasch analysis supported the unidimensionality of the HKCAS-T Cognition Scale. The scale differentiated among children of different ages, with older children achieving higher scores. The HKCAS-T Cognition Scale scores also correlated positively with the Cognitive Scale scores in the Cognitive Battery of the M-P-R. Internal consistency and test–retest reliability were both 0.98. **Conclusions**: The Cognition Scale of the HKCAS-T demonstrated strong psychometric properties and shows promise as an assessment tool for toddlers.

## 1. Introduction

According to the World Health Organization [1], developmental delay refers to limitations in body functions and activities arising from the interactions between health conditions and contextual factors (e.g., social structures, background, and attitudes). Such delays can hinder a child’s full participation in everyday activities, and environmental and personal factors may further impede functional development. Early identification and intervention for children with developmental delays are therefore important. These measures enable families to access appropriate services, empower both parents and children, and lead to improved developmental outcomes [2]. A longitudinal study has shown that health service provision incorporating early identification strategies, such as developmental screening and monitoring, significantly increases the likelihood of children receiving early intervention compared to settings without such strategies [3]. To implement such strategies effectively, culturally valid and reliable instruments are needed to assess child development across different ages.

In the Hong Kong Special Administration Region (SAR), China (hereafter referred to as Hong Kong), the Hong Kong Comprehensive Assessment Scales for Preschool Children (HKCAS-P) is available for children aged 3 years and 4 months to 6 years and 3 months [4]. The Wechsler Preschool and Primary Scale of Intelligence–Fourth Edition (Hong Kong) [WPPSI-IV (HK)] is available for children aged 4 years to 6 years and 11 months. Both tests are normed on Hong Kong children.

However, there are no locally normed instruments for toddlers. The Griffiths Mental Development Scales–Chinese (GDS-C) is normed on Chinese children (including those in Hong Kong and several major cities in mainland China), but it was based on the Griffiths Mental Development Scales Extended Revised (GMDS-ER) and not the updated Griffiths Scale of Child Development, Third Edition (Griffiths III). Other instruments, such as the Merrill-Palmer-Revised Scales of Development (M-P-R) [5], Bayley Scales of Infant and Toddler Development, Third Edition (Bayley-III), and Stanford Binet Intelligence Scale–Fifth Edition (SB-5)*,* are not normed for Hong Kong children.

To cater to the needs of toddlers, the pilot version of the Hong Kong Comprehensive Assessment Scales for Toddlers (HKCAS-T) was developed in 2020. It consists of four scales: Cognition, Language and Communication, Fine Motor, and Gross Motor. Three pilot studies [6,7,8] demonstrated preliminary evidence for the unidimensionality of the Cognition, Gross Motor, and Fine Motor scales. Scale scores differentiated between children of different age groups and between children with typical development and those with developmental delays. The next stage of psychometric evaluations for the HKCAS-T involves the examination of its concurrent validity and test–retest reliability, as well as re-examination of the previously investigated validity evidence using a more representative sample.

The present study focused on the validation of the Cognition Scale. Validity is defined as the extent to which a test measures what it purports to measure and can be evaluated in various ways [9]. Concurrent validity is established by investigating the association between the test scores and other related measures (usually the gold standard measures) [10]. In the present case, the HKCAS-T Cognition Scale score was compared with the scores of the Cognitive Scale in the Cognitive Battery of the M-P-R [5], with the expectation that there would be a positive correlation between children’s scores on these two tests. Another way to test for validity is to examine the relationship between the test score and relevant external variables [11]. In this case, the external variable was age group, with the expectation that older children would achieve higher scores.

In terms of the internal structure of a test, it is important to determine whether the test scores conform to the theoretical construct of unidimensionality or multidimensionality. In the present case, as the item scores in the Cognition Scale are to be summed to form a total score, unidimensionality must be confirmed. Rasch analysis was used to re-examine the measurement properties of the Cognition Scale in terms of unidimensionality, replicating the pilot study findings using a representative sample and exploring item reduction based on item goodness-of-fit and difficulty hierarchy. Infit and outfit statistics, point measure correlations, and principal component analysis of the residuals that remained after the extraction of the linear Rasch measure (PCA) were used to examine unidimensionality [12]. To examine the targeting of the Cognition Scale, Wright maps were used to examine the difficulty level of the items in relation to the ability of the children and identify redundant (or less sensitive or less favorable) items with similar difficulty levels. In addition to validity, reliability was assessed through internal consistency (KR-20) and test–retest reliability (intraclass correlation).

The present study differed from the pilot studies [6,7,8] in that it used a more representative sample recruited from 18 districts in Hong Kong (instead of 4 districts as in the pilot studies), included concurrent validity testing with the Cognitive Scale in the Cognitive Battery of the M-P-R [5], and incorporated a test–retest reliability component after a four-week interval. Additionally, the present study sought the possibility of reducing the test items without compromising psychometric properties.

The hypotheses were as follows:

1. HKCAS-T Cognition Scale scores would correlate positively with scores of the Cognitive Scale in the Cognitive Battery of the M-P-R.
2. HKCAS-T Cognition Scale scores would differentiate among children from different age groups, with older children obtaining higher scores.
3. HKCAS-T Cognition Scale scores would demonstrate acceptable test–retest reliability and exhibit the unidimensionality property.

## 2. Materials and Methods

### 2.1. Design

This study was a cross-sectional study.

### 2.2. Participants

The participants included 282 children, with 142 boys and 160 girls. They were divided into eight age groups, each covering a 3-month interval, with 30 to 38 children per age group. Participants were recruited through Maternal and Child Health Centers (MCHCs) of the Department of Health, Hong Kong SAR Government, from the 18 districts in Hong Kong [13]. Over 90% of newborn children in Hong Kong are registered with MCHCs. If there is more than one MCHC in the district, one MCHC was randomly selected to participate. Using the MCHC register as the sampling frame, in each MCHC, 16 children from each 3-month group (8 boys and 8 girls) were randomly selected. Based on MCHC records, children suspected of developmental problems were excluded. These included cases such as a referral for further assessment by MCHC doctors, referral for assessment by the Child Assessment Service (CAS), or children with a confirmed diagnosis from the CAS. In total, 7000 invitation letters were sent, and 969 parents gave consent for their children to participate. The response rate was 13.8%. Out of these 282 children, 41 were tested again 4 weeks after the initial assessment to examine the test–retest reliability.

The sample size was considered adequate for Rasch analysis, comparison of children across the eight age groups, and correlation with criterion tests in respective domains. For Rasch analysis, for high-stakes tests, a sample size of 250 is adequate [14]. In the present study of 282 children, the sample size is considered adequate. The sample size required for comparison across the eight age groups is 240 (power = 0.80, α = 0.05), with the assumption of a medium effect size. For correlation with the criterion tests in respective domains, the sample size calculation is based on the data on the construct and concurrent validity of the Australian Early Development Index (AEDI) and the Early Development Index (EDI) projects [15,16] where the correlation between the AEDI and EDI with the Peabody Picture Vocabulary Test (PPVT) and other cognitive measures in the validation studies is around 0.30. A sample size of 84 is needed (*p* = 0.80, alpha = 0.05) for a correlation of 0.30 [17]. For test–retest reliability, a sample size of 21 could provide 95% confidence intervals of a width of 0.20 for an intraclass correlation coefficient of 0.90 [18].

### 2.3. Measures

The Hong Kong Comprehensive Assessment Scale for Toddlers (HKCAS-T) was designed for Cantonese-speaking toddlers from 18 to 41 months [6,7,8]. Its pre-final version consists of four domains, namely, Cognition (83 items), Language and Communication (35 items on comprehension and 40 items on production), Fine Motor (50 items), and Gross Motor (37 items). The HKCAS-T was individually administered to all children by educational/clinical psychologists or medical practitioners experienced in the assessment of children. They have received standardized training in terms of test administration and scoring by the test developers prior to field testing. The field testers were aware of the age of the child being tested. All children were tested on all test items. The Cognition Scale was developed based on the Cattell–Horn–Carroll theory, which is consistent with commonly used assessment tools such as the Wechsler scales [19,20,21]. In the pilot study on the pre-final version of the Cognition Scale [6], the data supported both the 83-item and 77-item versions (6 items removed because of unsatisfactory goodness-of-fit statistics), with interrater reliability (Kappa) from 0.90 to 1.00 among field testers. The live assessment was video recorded, and a second rater scored the child’s performance by watching the video-taped session, blind to the scores of the live session, and the developmental status of the child in the live session.

The Cognitive Scale in the Cognitive Battery of the M-P-R [5] was used as the gold standard measure to test the concurrent validity of the Cognition Scale of the HKCAS-T in this study. The M-P-R is an updated developmental test that has been found to show good concurrent and predictive validity [22]. Moreover, it has been commonly used by local psychologists in the assessment of toddlers and young children. The Cognitive Scale in the Cognitive Battery of the M-P-R [5] was administered to one-third of the children by educational/clinical psychologists.

### 2.4. Procedures

Upon receiving the list of eligible children provided by MCHCs, a research officer randomly selected one boy and one girl from each 3-month group at each MCHC. Invitation letters were sent to the parents of the selected children, who were requested to complete and return a consent form. Upon receiving the consent form, a research assistant contacted the parents to arrange a time and venue for assessment.

All children were assessed on the HKCAS-T. To minimize the demands on the children, the sample was divided into three domain groups, with 92 children being assessed on the criterion test for Cognition Scale (Cognitive Scale in the Cognitive Battery of the M-P-R), 95 being assessed on the Reynell Developmental Language Scales—Cantonese [23], the criterion test for the Language domain, and 95 being assessed on the Peabody Developmental Motor Scales—Second Edition [24], the criterion test for both Gross and Fine Motor domains. Each of the three domain groups consisted of 9–13 children from eight age groups and 5–6 children from 18 geographical districts. The order of administration of the HKCAS-T and the criterion tools was randomized for each child. Suitably qualified professionals (e.g., psychologists, occupational therapists, physiotherapists, speech-language therapists) administered the appropriate criterion tool in their respective domains to the children.

After the first assessment, the HKCAS-T was re-administered to a convenience group of 41 children whose parents consented for their children to be assessed twice in 4 week intervals to establish its test–retest reliability.

### 2.5. Ethics

This study was approved by the Ethics Committee of the Department of Health, Hong Kong SAR Government.

### 2.6. Data Analysis

To examine the concurrent validity of the Cognition Scale of HKCAS-T, the correlation of the Cognition Scale scores with scores of the Cognitive Scale in the Cognitive Battery of the M-P-R was examined. Analysis of variance (ANOVA) was used to test whether the HKCAS-T Cognition Scale could differentiate children from different age groups. Rasch analysis was used to examine the unidimensionality and targeting of the HKCAS-T Cognition Scale, followed by exploring the possibility of deleting poorly performing items based on the results of the goodness-of-fit and item hierarchy. Test–retest reliability was examined using intraclass correlation. Internal consistency was evaluated using KR-20.

## 3. Results

The demographic characteristics of the participants are shown in Table 1.

### 3.1. Unidimensionality, Targeting, and Differential Item Functioning

For unidimensionality, the Rasch analysis results showed that the infit and outfit statistics of items 1, 3, 21, 22, 65, and 66 were outside the recommended range of 0.60 to 1.40. This was similar to the pilot study [6] where the infit and outfit statistics of four items (1, 21, 65, and 66) were outside the 0.60 to 1.40 range. After removing these six items, it was found that the infit and outfit statistics of item 52 were outside the recommended range. After removing this item, the infit and outfit statistics of items 2 and 23 were outside the recommended range. When these two items were removed, all infit statistics were within the recommended range, resulting in a 74-item version based solely on psychometric considerations. When the 74-item version was discussed with the group of professionals (psychologists and pediatricians) who developed the Cognition Scale, they suggested to keep items 2 and 3 (as these two items and item 4 are part of a three-hole formboard task), and further delete items 47 and 50 (these two are redundant with items on shape naming), as well as 59 and 60 (these items are considered too easy for toddlers), resulting in a 72-item version incorporating psychometric considerations and clinical judgment. With this reduced version, the infit statistics of all items except item 3 were within the recommended range. The point measure correlations of all three versions (i.e., the 83-, 74-, and 72-item versions) were positive.

PCA of the residuals was also used to examine the unidimensionality of the Cognition Scale. The criteria for unidimensionality are as follows: (i) the variance explained by measures must be over or equal to 40%; (ii) the variance explained by the first principal component of the residuals must be less than or equal to 15%; and (iii) the ratio of variance in measures to variance in the first principal component of the residuals must be at least 3:1 or higher [25]. The variances explained by measures of the 83-item version, 74-item version, and 72-item version were 56.9%, 56.7%, and 59.1%, respectively. The variances explained by the first principal component of the residuals of the 83-item version, 74-item version, and 72-item version were 2.6%, 2.9%, and 2.7%, respectively. Finally, the ratios of the variance explained by measures to variance in the first component of the residuals of the 83-item version, 74-item version, and 72-item version were 21.88:1, 19.55:1, and 21.88:1. The results met the criteria for unidimensionality [25].

The person reliability estimates of the 83-item and 72-item versions were 0.97 and that for the 74-item version was 0.96. The person separation estimates of the 83-item version, 74-item version, and 72-item version were 5.66, 5.19, and 5.28, respectively. The item reliability estimates of the three versions were 0.99. The item separation estimates of the 83-item version, 74-item version, and 72-item version were 11.18, 10.86, and 11.51. The infit and outfit statistics and point measure correlations of the three versions are shown in Table 2.

**Figure 1:**
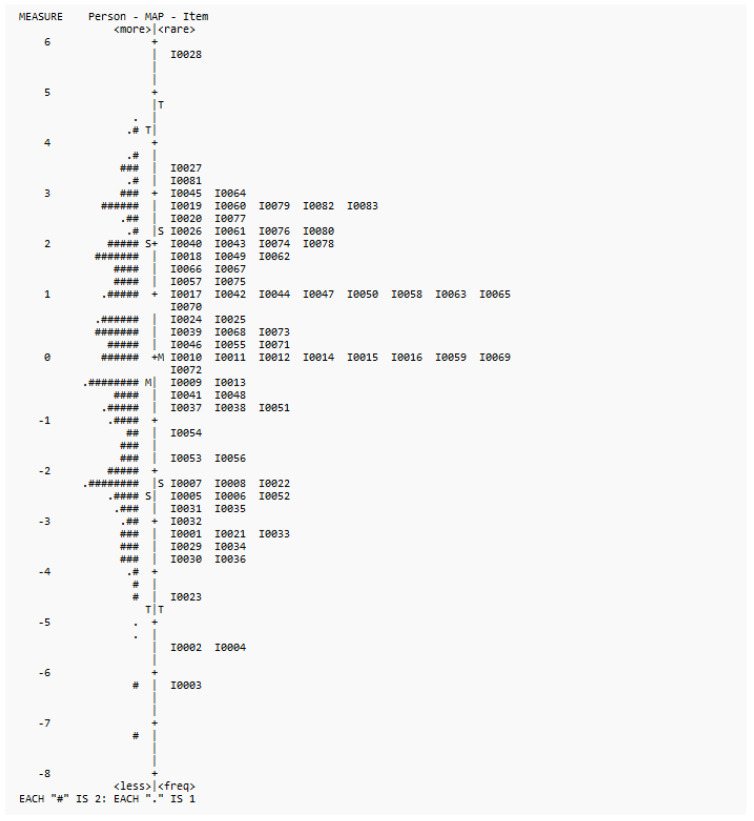
Wright map for 83 items.

**Figure 2:**
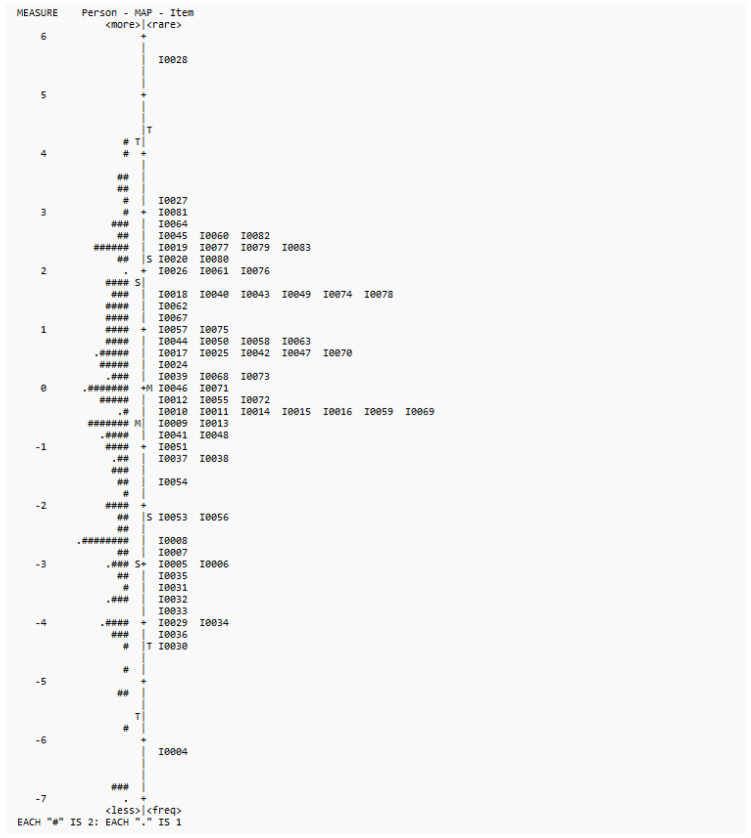
Wright map for 74 items.

**Figure 3:**
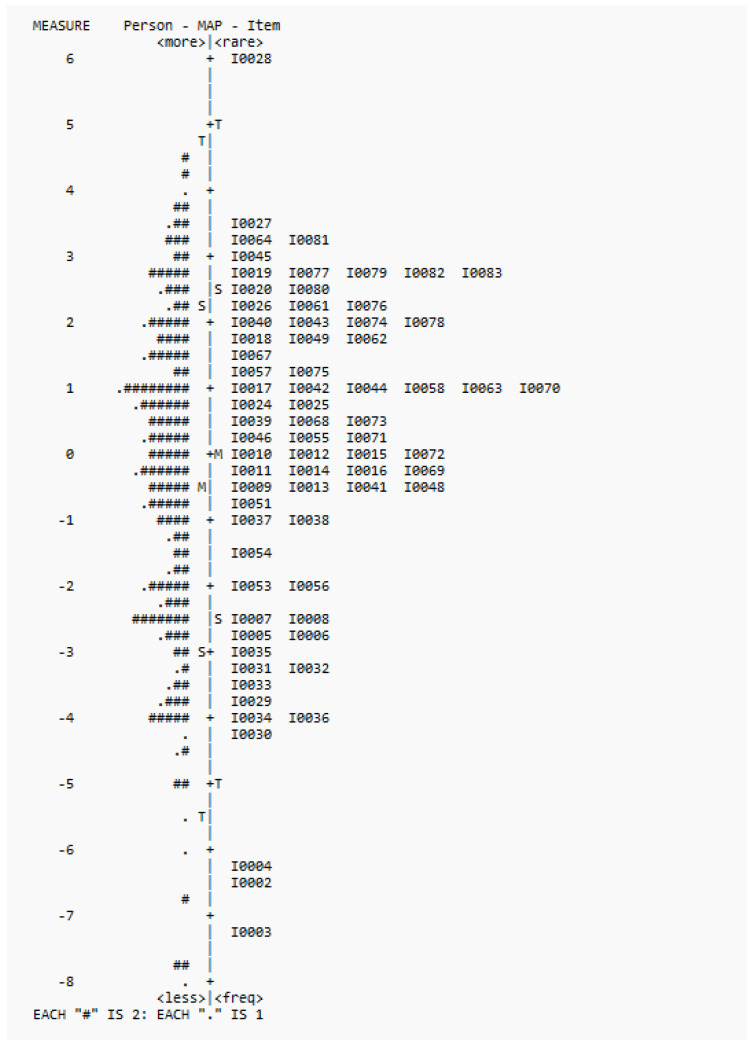
Wright map for 72 items.

In terms of targeting, Wright maps showed that all three versions could target the ability range of the participants, though there were fewer items at the upper and lower ends. The Wright maps of the three versions are shown in Figure 1, Figure 2 and Figure 3. Most of the deleted items (1, 21, 22, 23, and 52) fell into the lower end of the difficulty range. Some items (47, 50) were at the same difficulty range and were considered redundant by our experts. Items 60 and 66 were at the higher end, whereas item 59 was at the mean range.

For differential item functioning (DIF) by sex using Rasch analysis, the results indicated no item with statistically significant DIF by sex in the 83-item version, after Bonferroni adjustment for inflated alpha due to the large number of items. For the 74-item and 72-item versions, DIF was significant only for item 45 (naming shape), with boys attaining higher scores than girls. However, independent *t*-test results indicated no significant sex differences in the total scores of all three versions.

### 3.2. Validity Based on the Relationship with the Criterion Test and External Variable

In terms of the correlation of the Cognition Scale of the HKCAS-T with its criterion, the Cognitive Scale in the Cognitive Battery of the M-P-R, the correlations were around 0.70 for the three versions. When the correlations were analyzed by individual age groups, the correlations ranged from −0.01 to 0.82, with correlations of at least a medium effect size (above 0.40) for five of the eight age groups [26]. The details are summarized in Table 3.

For differentiation between different age groups, ANOVA results were significant for the 83-item version, *F*(7,274) = 68.22, *p* < 0.001; the 74-item version, *F*(7,274) = 68.12, *p* < 0.001; and the 72-item version, *F*(7,274) = 69.70, *p* < 0.001. A post hoc test (Scheffe) indicated that in all three versions, the test was able to differentiate among age groups, except the immediately adjacent age groups, in most cases. In all cases, children in the older age groups achieved higher scores than children in the younger age groups. The details are shown in Table 4.

### 3.3. Reliability

With regard to reliability, the KR-20 of the 83-item version, 74-item version, and 72-item version was 0.98. For test–retest reliability, the intraclass correlation of the 83-item version, 74-item version, and 72-item version was 0.98.

## 4. Discussion

### 4.1. Validity of the Cognition Scale

Hypothesis 1 on the correlation between the HKCAS-T Cognition Scale and scores of the Cognitive Scale in the Cognitive Battery of the M-P-R was largely supported. The overall correlations between the HKCAS-T Cognition Scale and the Cognitive Scale in the Cognitive Battery of the M-P-R were around 0.70 for all three versions. When analyzed by separate age groups, 62.5% of the correlations were at or above 0.40 for all three versions. The results were comparable with the correlation between the AEDI and EDI, which was around 0.30 [15,16]. The age-group-specific correlations were lower than those between the HKCAS-P and WPPSI-R, which were above 0.50 [27]. In the case of WPPSI-R, age-standardized scores were used, but in the Cognitive Scale in the Cognitive Battery of the M-P-R, only raw scores were used. An assessment of toddlers is likely to be more challenging than an assessment of preschool children because of the young age and shorter attention span of the former, and some haphazard responses among toddlers are not unexpected. Preschool children have all had some experience in a preschool setting where there is a demand for attention span and following instructions. In the complete sample, older children mostly obtained higher scores on both tests than younger ones, resulting in a positive correlation. Within individual age groups, with the small sample size, a low correlation may result due to the presence of one or two outlying scores [28]. In this age range, the development of children is variable.

Hypothesis 2 on the differentiation of age groups was largely supported. The HKCAS-T Cognition Scale could differentiate between different age groups, with older children attaining higher scores than younger children, though the differences might not be significant for every adjacent age group. The results were similar to the pilot study of the HKCAS-T Cognition Scale [6].

### 4.2. Unidimensionality and DIF of the Cognition Scale

For Hypothesis 3 on the unidimensionality of the Cognition Scale, Rasch analysis results provided support for its unidimensionality in terms of infit and outfit statistics, point measure correlations, and PCA results. For targeting, the items could target the ability range of children aged 18 to 41 months, though there were few items at both ends. The results were largely consistent with the pilot study results [6]. In terms of DIF, though there was one item with significant DIF values, this item was one of the most difficult items across the three versions (see Figure 1, Figure 2 and Figure 3). Also, considering the satisfactory overall measurement properties and validity of the three versions, and the non-significant difference by sex (*t*-test results) in terms of the overall scores, this item was retained. Overall, the DIF results suggest that the interpretability of the Cognition Scale is not significantly affected by the interaction between item and sex.

### 4.3. Reliability of the Cognition Scale

With regard to Hypothesis 3 on the reliability of the Cognition Scale, both the internal consistency and test–re-test reliability were above 0.90, indicating good reliability.

### 4.4. Limitations and Implications for Further Studies

There were some limitations in the present study. First, the sample size for correlations within individual age groups was less than required, though the sample size for overall correlations was adequate. In the correlation study, toddlers had to complete two tests, which may have been physically and mentally demanding for them. It was a demanding task to accumulate a sufficient number of toddlers who could complete two tests.

Second, the response rate was low and might not be very representative of the population of toddlers in Hong Kong. This study was started just before the beginning of the COVID-19 pandemic and continued throughout and beyond the pandemic. Parents might have been reluctant to take their young children out unless absolutely necessary during the pandemic. Nonetheless, the sample included children selected from all districts of Hong Kong.

Third, the sample excluded children with suspected developmental problems. However, the ability of the Cognition Scale to differentiate between children with typical development and children with developmental delay was demonstrated in the previous study [6]. A norming study is currently in progress, and the study aims to sample a portion (3%) with a confirmed diagnosis of developmental delay.

Fourth, the sample only included children registered with the public service (Maternal and Child Health Centers) but not children who were not registered with this public service.

Fifth, there were items with outfit statistics outside the recommended range, which were still included in the 74-item and 72-item versions. It is recommended that more attention should be given to infit statistics, as outfit statistics are not weighted and are easily influenced by outliers [29], and responses to items which are highly discrepant from a person’s ability, such as guesses and careless mistakes [30]. With toddlers, it is a challenging task to keep them motivated, cooperative and attentive throughout the test process, even though all the field testers were experienced in or trained to work with young children.

Sixth, the Wright maps indicated that there were fewer items targeting the upper and lower ends of the ability range. The scale might be less able to map the cognitive functioning of younger children with significant delay and older children who are more advanced in their development.

Seventh, the reliability estimates are high (approaching 1.00), indicating the possibility of item redundancy. At this early stage of test development, we chose to include more items in our investigation. We are aware that redundancy may limit efficiency. When the data from the norming study (mentioned above) become available, we will be able to further examine the data and discuss with the test developers how to reduce item redundancy if needed, while bearing in mind the need to preserve validity. With widespread use of the test in the future, we could be guided by additional data, accumulating clinical practice and judgment on the use of the test, to trim the test.

Eighth, although the field testers were experienced clinicians who had received training from the test developers, there was no fidelity check during field testing in this round of data collection. We were not able to conduct interrater reliability due to limitations in manpower resources.

Ninth, the field testers were aware of the age of the children they assessed. However, they were required to administer all test items to all children, regardless of age.

Finally, we did not examine predictive validity, which requires a longitudinal design for investigation in future studies.

## 5. Conclusions

The present study provided further evidence on the psychometric properties of the Cognition Scale of the HKCAS-T in terms of its concurrent validity, internal consistency, and test–retest reliability. The unidimensionality and targeting properties were also satisfactory. The Cognition Scale of the HKCAS-T is a promising instrument for assessing the cognitive development of toddlers in Hong Kong. The psychometric and measurement properties of the three versions (83-item, 74-item, and 72-item) were similar and satisfactory. The 72-item version is regarded as the preferred version as it is shorter and less demanding for toddlers, and its item selection is based on expert clinical opinion and statistical considerations. Though it is developed for Hong Kong toddlers, the test is potentially useful for the Chinese population in areas outside Hong Kong and for researchers interested in the development of toddlers.

## Figures and Tables

**Table 1:** Demographic characteristics of participants.

	18–20 Months (*n* = 30)	21–23 Months (*n* = 38)	24–26 Months (*n* = 35)	27–29 Months (*n* = 38)	30–32 Months (*n* = 35)	33–35 Months (*n* = 36)	36–38 Months (*n* = 36)	39–41 Months (*n* = 34)
Sex of child—boy	14 (46.7%)	20 (52.6%)	16 (45.7%)	19 (50.0%)	17 (48.6%)	19 (52.8%)	20 (55.6%)	17 (50.0%)
Sex of child—girl	16 (53.3%)	18 (47.4%)	19 (54.3%)	19 (50.0%)	18 (51.4%)	17 (47.2%)	16 (44.4%)	17 (50.0%)
Child’s education—no schooling	27 (90.0%)	26 (68.4%)	17 (48.6%)	16 (42.1%)	14 (40.0%)	10 (27.8%)	3 (8.3%)	5 (14.7%)
Child’s education—preschool/nursery	3 (10.0%)	12 (31.6%)	18 (51.4%)	22 (57.9%)	21 (60.0%)	26 (72.2%)	33 (91.7%)	29 (85.3%)
Mother tongue—Cantonese	29 (96.7%)	35 (92.1%)	33 (97.1%)	35 (94.6%)	31 (88.6%)	36 (100.0%)	36 (100.0%)	32 (94.1%)
Mother tongue—Mandarin	0 (0.0%)	1 (2.6%)	0 (0.0%)	0 (0.0%)	0 (0.0%)	0 (0.0%)	0 (0.0%)	0 (0.0%)
Mother tongue—English	1 (3.3%)	2 (5.3%)	1 (2.9%)	2 (5.4%)	4 (11.4%)	0 (0.0%)	0 (0.0%)	2 (5.9%)
Nuclear families	15 (50.0%)	18 (48.6%)	18 (54.5%)	19 (51.4%)	21 (60.0%)	25 (71.4%)	20 (57.1%)	21 (61.8%)
Extended families	14 (46.7%)	17 (45.9%)	14 (42.4%)	17 (45.9%)	12 (34.3%)	10 (28.6%)	13 (37.1%)	13 (38.2%)
Re-constituted families	0 (0.0%)	1 (2.7%)	0 (0.0%)	1 (2.7%)	0 (0.0%)	0 (0.0%)	0 (0.0%)	0 (0.0%)
Other types of families	1 (3.3%)	1 (2.7%)	1 (3.0%)	0 (0.0%)	2 (5.7%)	0 (0.0%)	2 (5.7%)	0 (0.0%)
Married	28 (93.3%)	36 (94.7%)	33 (94.3%)	34 (91.9%)	33 (94.3%)	34 (97.1%)	33 (91.7%)	32 (97.0%)
Separated/divorced/widowed	0 (0.0%)	0 (0.0%)	1 (2.9%)	3 (8.1%)	0 (0.0%)	0 (0.0%)	2 (5.6%)	1 (3.0%)
Single	2 (6.7%)	2 (5.3%)	1 (2.9%)	0 (0.0%)	2 (5.7%)	1 (2.9%)	1 (2.8%)	0 (0.0%)
Mother’s education—≤9 years	5 (16.7%)	2 (5.4%)	2 (5.7%)	5 (13.9%)	5 (14.3%)	6 (16.7%)	6 (16.7%)	5 (15.2%)
Mother’s education—>9 years	25 (83.3%)	35 (94.6%)	33 (94.3%)	31 (86.1%)	30 (85.7%)	30 (83.3%)	30 (83.3%)	28 (84.4%)
Father’s education—≤9 years	4 (13.8%)	4 (10.8%)	4 (11.4%)	4 (10.8%)	4 (12.1%)	6 (16.7%)	5 (14.3%)	2 (6.1%)
Father’s education—>9 years	25 (86.2%)	33 (89.2%)	31 (88.6%)	33 (89.2%)	29 (87.9%)	30 (83.3%)	30 (85.7%)	31 (93.9%)
Family income ≤HKD 29,999	10 (34.5%)	14 (37.8%)	8 (22.9%)	11 (30.6%)	13 (37.1%)	11 (32.4%)	15 (41.7%)	6 (19.4%)
Family income ≥ HKD 30,000	19 (65.5%)	23 (62.2%)	27 (77.1%)	25 (69.4%)	22 (62.9%)	23 (67.6%)	21 (58.3%)	25 (80.6%)
Mother’s length of residence in Hong Kong (years)	26.24 (11.85)	29.13 (12.27)	30.68 (10.90)	28.97 (12.11)	29.03 (12.20)	27.77 (12.02)	26.33 (12.60)	28.10 (13.64)
Father’s length of residence in Hong Kong (years)	36.21(7.64)	33.53 (14.32)	36.77 (10.87)	35.64 (10.84)	36.13 (5.99)	33.27 (12.74)	36.44 (7.73)	34.31 (10.66)
Number of siblings	1.50 (0.78)	1.47 (0.56)	1.57 (0.74)	1.76 (0.83)	1.74 (0.75)	1.75 (0.81)	1.56 (0.65)	1.64 (0.70)

Note: The 2022 median household income in Hong Kong was HKD 28,300 [13].

**Table 2:** Infit and outfit statistics and point measure correlations.

	83 Items			74 Items			72 Items		
Item	Infit	Outfit	Corr	Infit	Outfit	Corr	Infit	Outfit	Corr
1.	1.60	2.83	0.35	---	---	---	---	---	---
2.	0.69	0.97	0.39	---	---	---	0.60	2.14	0.44
3.	0.58	0.09	0.37	---	---	---	0.58	0.08	0.41
4.	0.71	0.64	0.41	0.68	0.95	0.42	0.65	1.21	0.46
5.	0.75	0.42	0.67	0.79	0.43	0.69	0.79	0.42	0.69
6.	0.91	0.53	0.63	0.98	0.59	0.64	0.97	0.60	0.64
7.	0.92	1.34	0.62	0.96	0.85	0.65	0.95	1.64	0.64
8.	0.82	0.49	0.67	0.86	0.50	0.69	0.86	0.51	0.69
9.	0.84	0.70	0.71	0.87	0.75	0.71	0.89	0.78	0.70
10.	0.93	0.75	0.68	0.95	0.78	0.68	0.97	0.84	0.67
11.	0.91	0.69	0.69	0.94	0.69	0.69	0.95	0.71	0.68
12.	0.89	0.69	0.69	0.91	0.69	0.69	0.93	0.72	0.68
13.	0.98	0.83	0.67	1.03	0.92	0.67	1.06	1.03	0.65
14.	0.95	0.76	0.67	0.98	0.78	0.67	0.99	0.81	0.67
15.	0.98	0.98	0.66	1.01	1.76	0.65	1.03	1.70	0.65
16.	0.90	0.75	0.69	0.92	0.84	0.69	0.94	0.87	0.68
17.	0.82	0.62	0.67	0.84	0.65	0.66	0.85	0.64	0.66
18.	0.72	0.72	0.63	0.73	0.79	0.62	0.72	0.79	0.61
19.	0.70	0.36	0.57	0.70	0.35	0.56	0.70	0.35	0.55
20.	0.76	0.84	0.56	0.77	0.89	0.55	0.77	0.90	0.54
21.	1.47	3.32	0.36	---	---	---	---	---	---
22.	1.43	2.23	0.47	---	---	---	---	---	---
23.	1.23	1.34	0.38	---	---	---	---	---	---
24.	1.13	1.05	0.59	1.16	1.11	0.58	1.18	1.17	0.58
25.	0.87	0.62	0.67	0.90	0.64	0.66	0.90	0.63	0.66
26.	0.87	0.55	0.56	0.89	0.56	0.55	0.87	0.55	0.55
27.	0.99	0.62	0.40	1.00	0.67	0.38	1.00	0.66	0.38
28.	0.97	0.21	0.19	0.98	0.22	0.18	0.99	0.24	0.17
29.	0.85	0.36	0.58	0.84	0.32	0.61	0.81	0.31	0.62
30.	0.88	0.37	0.52	0.92	0.37	0.55	0.90	0.34	0.57
31.	0.90	0.55	0.60	0.91	0.56	0.63	0.88	0.56	0.64
32.	0.87	0.76	0.59	0.86	0.84	0.62	0.84	0.83	0.63
33.	0.72	0.31	0.62	0.69	0.28	0.66	0.66	0.26	0.67
34.	0.86	0.36	0.56	0.88	0.34	0.60	0.85	0.31	0.61
35.	0.76	0.36	0.65	0.76	0.33	0.68	0.73	0.31	0.68
36.	0.73	0.33	0.58	0.75	0.31	0.60	0.72	0.27	0.62
37.	1.20	1.30	0.60	1.29	1.83	0.59	1.32	2.05	0.58
38.	1.18	1.14	0.61	1.27	1.39	0.61	1.29	1.45	0.60
39.	1.08	1.15	0.61	1.12	1.52	0.60	1.13	1.57	0.60
40.	1.02	0.73	0.55	1.06	0.77	0.54	1.07	0.77	0.53
41.	0.98	1.08	0.66	1.02	1.25	0.66	1.05	1.28	0.65
42.	1.00	0.71	0.63	1.02	0.73	0.62	1.05	0.75	0.61
43.	1.13	1.54	0.50	1.16	1.76	0.50	1.19	2.12	0.49
44.	1.03	0.99	0.60	1.05	1.10	0.59	1.08	1.17	0.59
45.	1.16	0.86	0.40	1.18	0.83	0.40	1.18	0.85	0.40
46.	1.11	0.99	0.62	1.17	1.15	0.61	1.20	1.31	0.60
47.	1.21	1.16	0.55	1.24	1.38	0.55	---	---	---
48.	1.22	1.17	0.60	1.29	1.39	0.59	1.34	1.55	0.58
49.	1.15	1.29	0.50	1.18	1.68	0.49	1.21	1.81	0.48
50.	1.24	1.16	0.53	1.29	1.25	0.53	---	---	---
51.	0.80	0.79	0.71	0.83	0.83	0.72	0.84	0.87	0.71
52.	1.27	1.69	0.51	---	---	---	---	---	---
53.	1.12	1.44	0.59	1.26	1.76	0.50	1.28	1.85	0.59
54.	0.91	0.84	0.68	1.00	0.96	0.68	1.03	0.98	0.67
55.	1.29	1.49	0.55	1.34	1.72	0.55	1.36	1.84	0.54
56.	1.03	1.22	0.62	1.12	1.90	0.62	1.14	3.55	0.61
57.	0.85	0.61	0.65	0.86	0.61	0.64	0.88	0.62	0.63
58.	0.90	0.93	0.64	0.91	0.92	0.63	0.93	0.95	0.63
59.	1.10	1.12	0.62	1.16	1.35	0.61	---	---	---
60.	1.02	1.92	0.43	1.05	2.76	0.41	---	---	---
61.	0.96	0.67	0.53	0.98	0.71	0.52	1.00	0.74	0.51
62.	0.99	1.16	0.56	1.01	1.53	0.55	1.03	1.80	0.55
63.	0.95	0.68	0.64	0.98	0.71	0.63	0.99	0.76	0.62
64.	1.02	0.54	0.44	1.04	0.55	0.42	1.03	0.55	0.42
65.	1.45	1.98	0.45	---	---	---	---	---	---
66.	1.57	2.79	0.37	---	---	---	---	---	---
67.	1.07	1.44	0.55	1.11	1.93	0.54	1.12	2.16	0.53
68.	1.01	1.02	0.63	1.07	1.18	0.62	1.09	1.23	0.62
69.	1.06	1.57	0.63	1.12	3.10	0.62	1.14	3.55	0.62
70.	0.93	0.67	0.64	0.96	0.70	0.63	0.97	0.73	0.63
71.	0.80	0.63	0.71	0.83	0.65	0.70	0.84	0.67	0.69
72.	0.75	0.57	0.73	0.80	0.65	0.72	0.81	0.70	0.71
73.	0.71	0.64	0.72	0.73	0.82	0.71	0.73	0.88	0.70
74.	1.00	0.88	0.54	1.03	0.90	0.54	1.04	0.98	0.53
75.	0.83	0.58	0.65	0.84	0.61	0.64	0.85	0.63	0.64
76.	0.93	0.63	0.54	0.95	0.65	0.53	0.94	0.65	0.52
77.	1.06	0.78	0.46	1.07	0.81	0.46	1.08	0.81	0.45
78.	0.94	0.62	0.57	0.95	0.63	0.56	0.96	0.64	0.55
79.	0.99	0.74	0.48	1.00	0.81	0.48	1.01	0.94	0.47
80.	1.04	1.20	0.49	1.07	1.79	0.48	1.08	1.83	0.48
81.	0.98	0.63	0.42	0.98	0.63	0.42	0.99	0.63	0.41
82.	1.19	1.05	0.40	1.22	1.13	0.39	1.23	1.14	0.39
83.	1.22	1.60	0.40	1.24	2.47	0.40	1.25	2.56	0.39

Note: Corr = point measure correlation.

**Table 3:** Correlations with MPR by age group.

Age Group	83-Item	*p* Level	74-Item	*p* Level	72-Item	*p* Level
18–20 months (*n* = 9)	−0.08	0.844	0.04	0.925	−0.01	0.987
21–23 months (*n* = 13)	0.21	0.500	0.18	0.552	0.20	0.512
24–26 months (*n* = 13)	0.45	0.121	0.47	0.102	0.48	0.094
27–29 months (*n* = 12)	0.46	0.130	0.47	0.123	0.51	0.092
30–32 months (*n* = 13)	0.81	<0.001	0.82	<0.001	0.82	<0.001
33–35 months (*n* = 12)	−0.06	0.846	−0.06	0.855	−0.07	0.835
36–38 months (*n* = 11)	0.53	0.095	0.54	0.088	0.49	0.129
39–41 months (*n* = 9)	0.54	0.133	0.49	0.176	0.40	0.292
All (*N* = 92)	0.69	<0.001	0.70	<0.001	0.70	<0.001

**Table 4:** Mean and standard deviation scores by age group.

Age Group	*n*	Mean	Standard Deviation	Group Differences
83-item version				
(1) 18–20 months	30	12.80	6.44	(1) versus (3), (4), (5), (6), (7), (8)
(2) 21–23 months	38	18.92	8.35	(2) versus (4), (5), (6), (7), (8)
(3) 24–26 months	35	28.00	11.79	(3) versus (1), (5), (6), (7), (8)
(4) 27–29 months	38	38.76	13.36	(4) versus (1), (2), (7), (8)
(5) 30–32 months	35	42.94	14.01	(5) versus (1), (2), (3), (7), (8)
(6) 33–35 months	36	47.61	12.82	(6) versus (1), (2), (3), (8)
(7) 36–38 months	36	56.28	18.68	(7) versus (1), (2), (3), (4), (5)
(8) 39–41 months	34	64.12	10.47	(8) versus (1), (2), (3), (4), (5), (6)
74-item version				
(1) 18–20 months	30	7.63	5.05	(1) versus (3), (4), (5), (6), (7), (8)
(2) 21–23 months	38	13.58	7.58	(2) versus (4), (5), (6), (7), (8)
(3) 24–26 months	35	21.74	11.17	(3) versus (1), (5), (6), (7), (8)
(4) 27–29 months	38	31.71	12.62	(4) versus (1), (2), (7), (8)
(5) 30–32 months	35	36.11	13.06	(5) versus (1), (2), (3), (7), (8)
(6) 33–35 months	36	40.14	12.33	(6) versus (1), (2), (3), (8)
(7) 36–38 months	36	48.67	17.75	(7) versus (1), (2), (3), (4), (5)
(8) 39–41 months	34	56.15	10.25	(8) versus (1), (2), (3), (4), (5), (6)
72-item version				
(1) 18–20 months	30	9.10	5.40	(1) versus (3), (4), (5), (6), (7), (8)
(2) 21–23 months	38	14.89	7.14	(2) versus (4), (5), (6), (7), (8)
(3) 24–26 months	35	23.03	10.57	(3) versus (1), (5), (6), (7), (8)
(4) 27–29 months	38	32.50	11.88	(4) versus (1), (2), (7), (8)
(5) 30–32 months	35	36.71	12.49	(5) versus (1), (2), (3), (7), (8)
(6) 33–35 months	36	40.58	11.60	(6) versus (1), (2), (3), (8)
(7) 36–38 months	36	48.39	16.71	(7) versus (1), (2), (3), (4), (5)
(8) 39–41 months	34	55.68	9.66	(8) versus (1), (2), (3), (4), (5), (6)

## Data Availability

The data presented in this study are available on reasonable request from the authors. The data are not publicly available due to privacy, ethical and funding restrictions.
